# Defence of the Ontario Veterinary College

**Published:** 1901-03

**Authors:** J. P. Foster

**Affiliations:** Selby, S. D.


### CORRESPONDENCE.

### DEFENCE OF THE ONTARIO VETERINARY COLLEGE.

EDITOR JOURNAL OF COMPARATIVE MEDICINE:

DEAR SIR: During the past year I have noticed a number of articles in both the JOURNAL and the *American Veterinary Review* in which the Ontario Veterinary College has been held up to ridicule. However, it is a significant fact that invariably the attack has been made by someone connected with one of the rival veterinary colleges. As an alumnus of the Ontario Veterinary College I hope I may be pardoned for venturing to make a few remarks. It would be very foolish for me to contend, and in fact is contrary to my belief, that, everything else being equal, an education acquired in a two-year college is as thorough as one gained in a three-year school; but I know it to be a fact that in some of these three-year schools there is little, if any, more work accomplished in three sessions than is mastered in two terms at the Ontario Veterinary College, by reason of shorter school hours, failure of professors of different branches to put in an appearance for several days (possibly weeks), and a general "go-as-you-please" style.

In the *American Veterinary Review* for March, 1900, Professor Williams, of the New York State Veterinary College, Ithaca, N. Y., refers to the well-known fact that students of the Ontario Veterinary College are required to pass the intervening time between their junior and senior terms with a qualified practitioner. In the article referred to Professor Williams does not eulogize the Ontario Veterinary College for this requirement; on the contrary, he condemns it on the theory that the veterinarian with whom the student practices may not be competent. This contention may hold good in some cases, but it is an arrangement that has produced some of the best-known and most capable veterinarians in the United States and Canada to-day.

The catalogues or announcements of several of the newer schools that have sprung up in this country during the past few years are really beautiful specimens of the printer's art, embellished in colored inks and embossed covers and filled with lengthy and detailed descriptions of the different branches taught, and although the attempts at ultra-professional terms are, to say the least, ludicrous, they are quite interesting reading. Compare one of these "catalogs" with the severely plain Ontario Veterinary College announcement and the Toronto book suffers by comparison (in looks), but on turning to its list of graduates the reader will notice scores of names that have contributed to the making of veterinary history on this continent during the past thirty years. Little attempt is made in the Ontario catalogue to enumerate the many important positions its graduates hold or have held, which is also a contrast to those more elaborate books in which the graduates' names are fortified with asterisks and other signs and underlined in "fine print," stating that they hold "this or that" petty job; and I have in my possession a catalogue that proudly proclaims, among the many other achievements of a certain graduate, that he is ex-county assessor.

Graduates of the Ontario Veterinary College hold some of the most responsible veterinary positions in the United States; several have received appointments as State veterinarians within a year, in addition to those already holding this important office in other States. Graduates of the Ontario Veterinary College are members of the faculty or instructors at the present time at the following veterinary schools, viz.: New York State Veterinary College, Ithaca, N. Y.; Veterinary Department, Ohio State University; Veterinary Department, Iowa State College, Ames, Iowa; Kansas City Veterinary College; United States College of Veterinary Surgeons, Washington, D. C.; Grand Rapids Veterinary College; Indiana Veterinary College, and McKillip Veterinary College.

The last-named college has on its faculty eight veterinarians. Of this number four are Ontario graduates, three are graduates of the home school, and one is a graduate of the Chicago Veterinary College at the time it was a two-term school. Now, as the Ontario Veterinary College graduates largely formed the foundation of this school in 1894 and taught the students who have since graduated and become members of the faculty, the question that presents itself to me is, Is not this school pretty strongly Toronto after all? It is known to the writer that the McKillip College, as late as one year ago, was very desirous of obtaining the services, as a member of its faculty, of a certain graduate of the Ontario Veterinary College. They were unable to secure him, however. The gentleman referred to is conceded to be one of the finest comparative anatomists in America, and would add prestige and dignity to the staff of any school with which he might be in any way connected.

I recently formed the acquaintance of a veterinarian who, after the formalities of an introduction were gone over, asked the question, What school are you from? The expression of pity that passed over his face upon my informing him that I was an Ontario graduate was indeed laughable. Upon further conversation it developed that his impressions in regard to the Toronto school had been gained almost entirely from remarks made in the veterinary journals. He seemed very proud of his alma mater and his superior veterinary education. He is a graduate of what is now a three-year school, but at the time of his graduation was a two-year school, and in its announcement issued at that time stated that "the session begins in October and ends the latter part of February." Think of it! two terms of less than five months each! And because this school, which is one of the oldest and best-known institutions of its kind in the East, has since extended its course to three terms of six months each, some of its graduates under the old regime of the type of my friend mentioned complacently regard themselves as shining examples of "higher veterinary education."

Dr. Stalker, Dean of the Veterinary Department, Iowa State College, Ames, Iowa, is a graduate of the Ontario Veterinary College. The Ames school has probably turned out as many high-class men in proportion to its total number of graduates as any school in the country. Among its graduates are Dr. Stewart, Dean of the Kansas City Veterinary College, and it must be apparent that the "old Ontario Veterinary College" is "at the bottom," and has been instrumental, either directly or indirectly, in producing much that is good in the veterinary line to-day in North America. Graduates of the Ontario Veterinary College residing in those States (and in Manitoba) where veterinarians from two-year schools are barred from practice have found it necessary to attend a third year in some one of the three-year colleges, and, peculiar as it may seem to some, they often at graduation capture first prize over those more fortunate students who have had the superior (?) advantage of having passed their whole period of professional study in the three-year school.

This has happened at the New York State Veterinary College, Ithaca, N. Y., and appears to be occurring with astonishing regularity for several years at McKillip. Perhaps Dr. Merillat can explain why it is that the boys from a school where, as a student himself, he says he was told that "the veterinarian who practised horse dentistry belonged to the same category as lightning-rod agents," can come over to his school and "walk off" with first prize in both the regular and post-graduate course and the medico-chirurgical course, as did Drs. Rutledge (Ontario Veterinary College, 1898) and West (Ontario Veterinary College, 1895) last spring and as did Dr. Lawley (Ontario Veterinary College, 1898) in 1899. Is it possible that the education acquired at Toronto had anything to do with the showing made at McKillip?

I am an American citizen and always have been. I am not "booming" Canadian institutions, neither am I entering a plea against the cause of "higher education;" but the inconsistencies of certain "boomers" and "rooters" in the employ of a few much-advertised veterinary schools as well as the sneers of two-year graduates of present three-year schools appear to me very ridiculous. I went to the Ontario Veterinary College of my own free will and because I wanted to go there. If it becomes necessary for me to take a third year in some three-year school, in order to lawfully practice, I shall probably do so; but I hope I shall not feel called upon to belittle my alma mater at any future time, as I should feel that I was following the example of "the dirty bird who befouled its own nest."

Respectfully yours,

J. P. Foster, V.S.

P. S.—Since writing the above I notice that changes have been made in the faculties of some of the colleges mentioned.

J. P. F.

Selby, S. D., January 15, 1901.

